# Monitoring aerial insect biodiversity: a radar perspective

**DOI:** 10.1098/rstb.2023.0113

**Published:** 2024-06-24

**Authors:** Silke Bauer, Elske K. Tielens, Birgen Haest

**Affiliations:** ^1^ Swiss Federal Institute for Forest, Snow and Landscape Research, 8903 Birmensdorf, Switzerland; ^2^ Swiss Ornithological Institute, Sempach, LU 6204, Switzerland; ^3^ Institute for Biodiversity and Ecosystem Dynamics, University of Amsterdam, 1090 GE Amsterdam, Noord-Holland, The Netherlands; ^4^ Department of Environmental System Science, Federal Institute of Technology (ETH), 8092 Zürich, Switzerland; ^5^ School of Biological Sciences, University of Oklahoma, Norman, OK 73019-0390, USA

**Keywords:** aeroecology, radar, migration, monitoring, biodiversity

## Abstract

In the current biodiversity crisis, populations of many species have alarmingly declined, and insects are no exception to this general trend. Biodiversity monitoring has become an essential asset to detect biodiversity change but remains patchy and challenging for organisms that are small, inconspicuous or make (nocturnal) long-distance movements. Radars are powerful remote-sensing tools that can provide detailed information on intensity, timing, altitude and spatial scale of aerial movements and might therefore be particularly suited for monitoring aerial insects and their movements. Importantly, they can contribute to several essential biodiversity variables (EBVs) within a harmonized observation system. We review existing research using small-scale biological and weather surveillance radars for insect monitoring and outline how the derived measures and quantities can contribute to the EBVs ‘species population’, ‘species traits’, ‘community composition’ and ‘ecosystem function’. Furthermore, we synthesize how ongoing and future methodological, analytical and technological advancements will greatly expand the use of radar for insect biodiversity monitoring and beyond. Owing to their long-term and regional-to-large-scale deployment, radar-based approaches can be a powerful asset in the biodiversity monitoring toolbox whose potential has yet to be fully tapped.

This article is part of the theme issue ‘Towards a toolkit for global insect biodiversity monitoring’.

## 1. Introduction

Trillions of insects use the airspace for key activities of their life cycle, such as daily foraging movements and seasonal migrations. Their movements link otherwise separated habitats, communities and ecosystems, and have implications for various ecological processes such as nutrient transfer, pollen dispersal and gene flow, food web interactions and pathogen dynamics [1,2]. Insect movements also provide services, e.g. pollination, seed-dispersal, and pest control; and disservices, e.g. pathogen dispersal and agricultural damage that are relevant to human agriculture, economy and health [3]. However, in the current biodiversity crisis, many insect populations have alarmingly declined [4–6] raising concerns among scientists and the public alike. The United Nations' Convention on Biological Diversity (CBD) is the established international legal instrument for the conservation of biological diversity and for actions that lead to a sustainable future (https://www.cbd.int). Biodiversity monitoring importantly contributes to this goal by providing an assessment of the current state of biodiversity, its changes over time, its response to (changes in) biodiversity drivers and management actions.

Over the past years, much effort has been put into standardizing biodiversity monitoring schemes that provide harmonized observations and regular, timely data on biodiversity change [7–9]. Consequently, a set of classes of essential biodiversity variables (EBVs) has been defined that can deliver multi-purpose, long-term biodiversity information at various scales: genetic composition, species populations, species traits, community composition, ecosystem structure and ecosystem function [10]. The power of the EBV framework lies in the multiple dimensions of biodiversity change that these variables capture, their complementary scope, and generalizability across systems. EBVs specifically harness remote sensing approaches for spatially continuous sampling that can be repeated at the same locations or regions at shorter (1–5 years) or medium (10–50 years) time scales.

**Figure 1.**
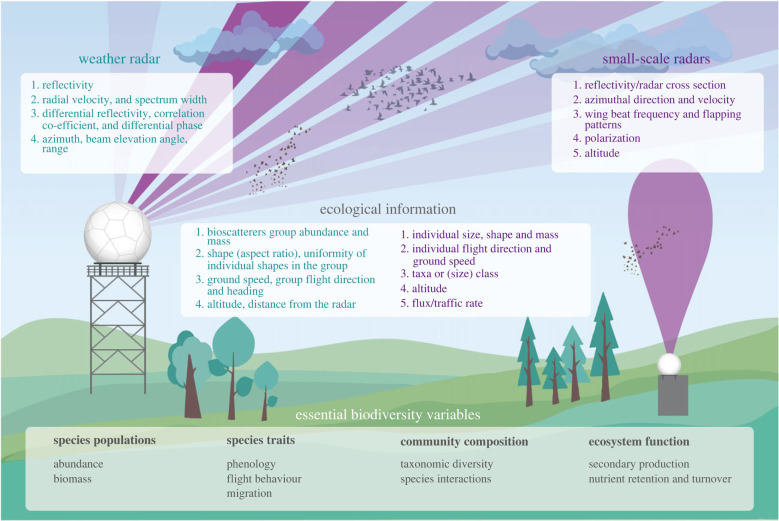
Principle of weather (left) and small-scale (right) radars, the ecological information that can be derived from them and how this ecological information contributes to several essential biodiversity variables.

Insects remain notoriously difficult to monitor owing to their small body sizes [11], enormous numbers, movements at large scales and heights, and often nocturnal and cryptic behaviour [1,2,12,13]. Remote sensing technologies such as radar are increasingly employed as they can provide detailed information on intensity, timing, altitude, and spatial scale of aerial mass movements for a broader range of taxa and all individuals passing through the sensor's measurement range (figure 1). Furthermore, as most radar systems are automated, they monitor the aerial environment continuously and provide a quantification of animal fluxes and flight behaviours at unprecedented detail and scales. Radars have been used in ecological research for decades, but improved classification algorithms and technical advances as well as data-sharing have greatly broadened the range of applications [14–16]. Although radars do not always provide taxon-specific data, they can contribute to the EBVs: (i) ‘species populations’ by quantifying abundances and distributions of groups of species and their variability over space and time; (ii) ‘species traits’, specifically phenology and movement behaviour by identifying flight behaviour and the timing and routes or migrations; (iii) ‘community composition’ by characterizing the composition and diversity of the community of aerial organisms and their interactions; and (iv) ‘ecosystem function’ by estimating secondary production, nutrient retention and turnover and disturbance regimes.

**Figure 2.**
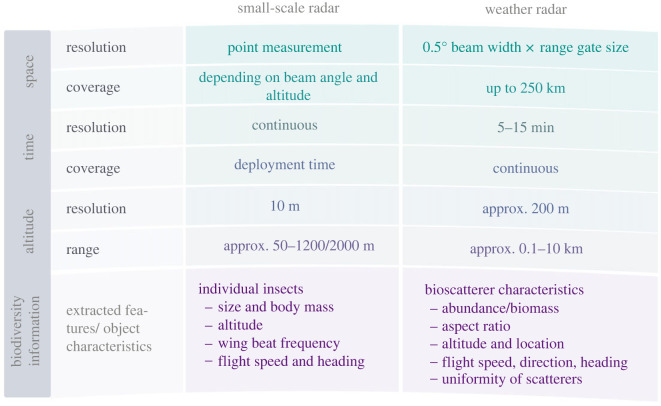
The main radar systems currently used for insect studies—small-scale biological and large-scale weather surveillance radars—and their main characteristics relevant for insect biodiversity monitoring. Please note that the specifications given are exemplary, only indicate the orders of magnitude and may differ for different models.

Various radar systems exist—from specialized small-scale radars to large-scale weather radars—that are largely complementary in spatio-temporal and taxonomic resolution and coverage (figure 2). While small-scale radars (e.g. vertical looking radars) can identify individual animals and characterize their body shape, size, wing beat frequency and other individual characteristics, they only monitor smaller spatial areas [17]. By contrast, weather surveillance radars (WSR) survey the atmosphere above many of the world's large landmasses and are therefore often organized in continental networks (NEXRAD in the United States or OPERA in Europe [18]), covering regions of several hundreds to many thousands of square kilometres. They also detect biological targets, albeit at coarser spatial and taxonomic resolution, and owing to their continual operation, they have great potential to be employed as a standardized monitoring system of aerial biodiversity [19,20]. In the following, we briefly characterize two major radar types that have been used in insect monitoring, synthesize their contributions to EBVs and outline challenges and opportunities in tapping the full potential of radars for standardized (insect) biodiversity monitoring.

## 2. Characterization of radar types used for insect monitoring

The general principle of radars consists of emitting an electromagnetic beam, of which objects in the air bounce back an echo, which is then received by a receiver. The radiated beams are focused on the airspace of interest through an antenna; and position and motion of the antenna determine the scanning strategy: in ‘fixed-beam sampling’, the beam is directed in a set direction while in ‘azimuth scanning’ the beam is swept continuously in azimuth (i.e. through 360°) at a succession of elevation angles [13,21]—with consequences for spatial and temporal coverage and resolution. The radar systems that have been used in insect research differ in technology and scanning strategy. Consequently, they differ in the variables they return for targets and thus, in their capacity to differentiate between insects and other aerial organisms, and between different groups of insects (figure 1) [21,22]. Dedicated algorithms have been developed to extract biological information from radar signals. They use various radar products to separate signals generated by animals in the air from signals from other sources such as precipitation and ground echoes or other anomalous propagation. In the early years of radar entomology, scanning radars were used to investigate the migration ecology of many insect taxa, predominantly pest species [22,23]. Here, we focus on two wide-spread, complementary radar systems that are currently predominantly used in insect studies—small-scale (vertical-looking) biological and large-scale weather radar. Although a variety of other radar types exists, e.g. frequency-modulated continuous wave radar [24], cloud radar [25,26], wind profiler [27] and airborne doppler radar [28], these have only sporadically been employed in insect studies and are therefore not discussed further.

Small-scale (vertical-looking) biological radars use fixed-beam sampling with a beam angle of only a few degrees, which increases beam intensity, detectability of weakly reflecting targets, spatial resolution and altitudinal range (figure 2). While the beam could be pointed in any direction, in practice it is always pointed upwards, albeit sometimes with a small angular offset [17,22,27,29]. These radars record the movements of single individuals passing through the radar beam and the returned echo contains information on an individual's trajectory (height, displacement speed, alignment), wing beat frequency and radar cross section [17]. Morphological parameters of individuals, including mass, body length and shape are estimated from temporal, angular and polarization variation in the radar cross section (figure 2) [17,21,30]. In contrast to weather radars that measure the volume density of animals in the air (see below), vertical looking radars register insect movement fluxes (often referred to as migration traffic rates), i.e. the number of insects passing through a virtual unit area of air, often standardized to the number of insects per kilometre and hour. When the radar system, however, also allows estimation of insect ground speeds, these fluxes can be converted to volume densities [31].

Small-scale radars have a long history of technological developments and customization. The models in insect studies primarily use the zenith-looking linear-polarized conical scan (ZLC) configuration [21]. Variations in technological designs mostly pertain to beamwidth and, more recently, wavelength, both of which influence (altitudinal) range, spatial resolution, and the minimum size of insects that can be detected. X-band radars with a wavelength of 8 to 12 GHz or 2.5 to 3.75 cm are the most common. Because of the recovery time of the pulse, a ZLC radar can typically not record targets or flight activity in the first tens of meters above. On the more practical side for longer-term operation, small-scale vertical-looking radars have comparatively few moving parts, which reduces service and maintenance required.

Commercially available models of small-scale biological radars are equipped with software to automatically classify objects into non-biological (e.g. clutter and rain) and several biological groups (e.g. insects, birds or subgroups of birds such as passerines and waders), based on a reference dataset with known identities (e.g. [32] for the Birdscan MR1). For the model with the currently broadest distribution, the Birdscan MR1, the R-package ‘birdScanR’ converts raw object counts into standardized measures of aerial activity, i.e. migration traffic rates and density [33].

Weather surveillance radar sweep a highly focused beam (typically about 0.5–1°) azimuthally through several elevation angles, resulting in relatively slowly updated (about 5–10 min) yet three-dimensional coverage of a large amount of airspace (figure 1). Consequently, they do not provide information on individual insects but for all scatterers in the entire range gate (i.e. the size of a single pulse volume at a given distance, which varies between systems and increases with distance from the radar). As many animals can be in the same range gate, echoes frequently originate from a mix of bioscatterers, which complicates interpretation. Consequently, weather radar data cannot directly estimate individual morphological parameters, but targets need to be classified from radar products, i.e. power of the returned signal, its phase, and the difference between these in horizontal and vertical polarizations (see [34] for details on these characteristics). Based on the radar output, scatterer size, aspect ratio, orientation, variation in orientations and target height can be estimated, which is sufficient to at least differentiate insect scatterers from vertebrates and precipitation [34,35].

In the past years, many weather radar systems have been updated to dual-polarization (dual-pol), where signals are sent out and returned in both vertical and horizontal planes. Because reflections from elongated objects like insects vary considerably between vertical and horizontal polarization, dual-pol radars are much better suited to identify mass movements of insects than single-pol radar. Common products from dual-pol radar include the power of the returned signal (i.e. reflectivity), the ratio between horizontal and vertical reflectivity (differential reflectivity), the phase shift between horizontal and vertical signals (differential phase), displacement speed (radial velocity), the variance of speeds within a volume (spectrum width), and the similarity of horizontal and vertical signals in a volume (correlation coefficient) [34]. These products are provided for every range gate, alongside information on the range gate location and height. Weather radars can operate at various bands. The most common types are X-band (3 cm), C-band (5 cm) and S-band (10 cm) radars. Radars with smaller wavelengths are more sensitive and better able to detect small particles such as insects, but this comes at a cost of a smaller range. Radars with longer wavelengths are better suited for surveillance at large extents but have lower precision for small targets. These types provide distinct trade-offs in their use for insect monitoring, and researchers must consider the consequences of the available weather surveillance radar type for their study system. Alternatively, integrating data from radars operating at different wavelengths (e.g. weather radar operating at S-band and scanning entomological radar at X- band) can optimize this trade-off [36].

Taxonomic resolution of weather radar data is limited because the data products provide information across all targets within a range gate volume, rather than quantifying a single insect target. As a result, weather radar data are commonly interpreted when a single taxon dominates in the radar beam, e.g. during mass movement of mayflies [37], grasshoppers [38] or pest species [39]. Biological information can be extracted from weather radar signals using the R-package ‘bioRad’, which differentiates animal (bird) echoes mainly based on correlation coefficient and radial velocity [40,41]. To distinguish insect signals from vertebrates, several methods have been developed, e.g. based on standard deviation of radial velocity [41,42], a combination of differential reflectivity and copolar correlation coefficient [43], or a combination of air speed and radial velocity [35]. Once the identity of targets in the radar beam is determined, the radar cross section, or the degree to which an animal reflects electromagnetic waves of a given wavelength, can be used to convert the returned signal to a density of individuals [44].

All radars inherently only observe insects when they are in the air and above a certain altitude (figure 2). However, the differences between radar types in scanning strategy, wavelength, power, and other characteristics have consequences for how and which insects are detected and thus, for which ecological questions they are best suited. For example, fixed-beam radars measure fluxes of insect movement, while scanning radars measure density within a given air volume. Small-scale radars have higher precision as they observe individual insect movements, while weather radars observe all insects in the range gate volume. Small-scale radars have higher resolution but a narrower spatial coverage. The radar's sensitivity to targets of different sizes also depends on radar type, wavelength and power (see above), but small-scale radars operate best with medium-sized and larger sized insects such as noctuid butterflies, hoverflies or grasshoppers and locusts, that are well within their measurement range and have consequently been the most targeted insect group. Small insects are generally harder to detect with small-scale radar and consequently, less commonly studied. By contrast, weather radars typically lack data on morphology or wingbeat frequency, but they are not limited by target size and can observe any insect signals [45].

## 3. Current state of radar-based biodiversity monitoring of insects

Although radar-based approaches have long been deemed to be unsuitable for traditional biodiversity monitoring owing to their inability to identify species, radars have been employed for investigating aerial insect movements quite extensively. In the following, we synthesize radar-based studies targeting aerial insects, insect migration (using the broader definition of migration entomologists that includes all radar-observed movements within the migration continuum [46]), and related topics in terms of the contribution they could make to EBVs.

### (a) Essential biodiversity variables class ‘species populations’: abundance and biomass estimates

Insects surpass other aerial fauna in terms of abundance and biomass, yet quantifications of aerial insect abundance have for long mostly relied on estimates from visual observations, e.g. daytime, near-surface counts [47], ground [48] or aerial trapping [26,49]. Radar approaches for quantifying insect abundance and biomass at larger spatial and temporal scales over various altitudes are still relatively rare, but a few notable exceptions exist: in one of the first such studies, abundances of diurnal and nocturnal aerial insects of three size-classes were measured with small-scale radars over the southern UK over several years [50]. This resulted in an estimated annual mean of 3.37 trillion insects that migrate over the UK, primarily during the daytime. Combining small-scale radar and aerial trapping showed that most migrating insects (greater than 99%) were small insects, making up 81% of biomass. Overall, the aerial insect biomass flux consisted of 3200 tons of biomass, which exceeds the mass of songbirds departing the UK for Africa each autumn sevenfold.

Filtering small-scale radar data to match body mass and shape (based on reflectivity) and diurnal flight timing to migratory hoverflies (*Episyrphus balteatus*), Wotton *et* *al*. [51] quantified the numbers and biomass of migrating individuals over a 10 year study period. Over their 300 km wide study region, this totalled a mean of 2.66 billion hoverflies per year with an average biomass of 55.5 tons or a density of 38 000 km^−2^—exceeding the earlier records of largest migrations of silver Y moths (*Autographa gamma*) 10-fold [52] or 70-fold the number of painted lady butterflies (*Vanessa cardui*) [53].

Early studies of insects with weather radar have mainly focused on quantifying insect aerial biomass at single radars and were restricted to reporting reflectivity from insect targets rather than abundance [54]. With advances in electromagnetic modelling of insect radar cross sections [37,44], reflectivity can be converted into abundance or biomass estimates and phenomena such as mass insect emergences can be quantified at scale. For example, mayfly (*Hexagenia* sp*.*) emergence from Lake Erie has been estimated to result in a flux of up to 87.9 billion individuals moving across the water–air interface in a single night [37]. In a study on macro-scale responses to artificial light using weather radars, an outbreak of grasshoppers (Orthoptera, predominantly pallid-winged grasshopper [*Trimerotropis pallidipennis*]) was recorded, that peaked at approx. 45 million individuals with 30.2 metric tons of biomass within a 175 km radius of the Las Vegas radar [38]. In the contiguous USA, weather radar data have recently provided continental scale estimates of insect density, indicating that continental scale temperature drives variation in aerial insect density across biomes [55].

Continuation of radar-based monitoring will result in increasingly longer time series of insect abundance, providing a unique opportunity to identify changes in numbers and distributions in relation to environmental and socio-economic variables. A few recent studies have shown stable temporal patterns in aerial biomass and abundance over the preceding decade [50,51]. Moreover, assessing changes in insect abundance and biomass is contingent on establishing location-specific historic baselines [56], and archives of historic weather radar data are uniquely positioned to facilitate this. Given the accelerated use of small-scale radars and ongoing advances in tools to analyse insect presence in weather radar, large volumes of data are becoming available to anchor and accelerate our understanding of abundance and biomass EBVs.

### (b) Essential biodiversity variables class ‘species traits’: phenology, flight behaviour and migration

Radars are uniquely suited to the study of aerial movement and migration because most migrations take pace above eye level, at altitudes from several tens of meters up to a few kilometres [1,48,57]. Some larger insects such as dragonflies (Odonata) and butterflies (Lepidoptera) migrate close to the ground, but most migrations take place at higher altitudes, including those of smaller and medium-sized insects, such as noctuid moths (Noctuoidea) and hoverflies (Syrphidae). Owing to the limits set by radar designs and measurement characteristics, most studies of migration patterns and flight altitude, behaviour, direction, orientation and phenology pertain to intermediate and large sized insects.

Aerial insects show distinct diel activity periods [58]. Diurnal migrants appear in radar signal around mid-morning and end activity in the late afternoon; nocturnal migrants take off *en masse* at dusk and fly throughout the night. Using a network of small-scale radars for monitoring at large spatial extent, Haest *et al*. [59] demonstrate the surprising consistency of these diel activity patterns along a European gradient from southern France to Finland.

The directed movements of insects indicate seasonally preferred directions. For instance, noctuid moths (*A. gamma*, silver Y) arrive in spring in the UK to colonize temporary summer breeding grounds, after having travelled at altitudes of 200–1000 m above ground over approximately 300 km per night. As this species cannot survive the winter conditions in northern latitudes, it migrates to its winter-breeding grounds in North Africa and the Middle East in autumn [52]. Similarly and also using small-scale radar, Hu *et al*. [50] showed consistently northward mass migrations in spring and southward migrations in autumn across the southern UK. Migration intensity correlated with surface and higher-altitude wind directions, and migration takes place on days with winds in favourable directions, suggesting that insects actively choose a time for take-off that optimizes transport in a specific direction [49,60]. Noctuid moths and hoverflies select seasonally favourable winds but this selectivity is greater in spring when preferred winds are more common. During autumn, migrant headings are clustered tightly, indicating a stronger need to orientate in a seasonally preferred direction [61]. The phenological patterns of directional migration flights were less clear-cut in another small-scale radar study, which showed peaks in spring, summer and autumn for diurnal migrants but only an autumn peak for nocturnal migrants [29].

Some migrations are associated with the passage of fronts, such as moth migration in spring in southeastern Australia [62]. Transport is also enhanced by active flight, e.g. for noctuid moths, displacement speeds are commonly 4–6 m s^–1^ faster than the wind vector, indicating that insect-powered flight increases migration distances by 40% [63]. Active choice of flight height can also optimize transport; insect migrants commonly concentrate in well-defined horizontal layers. These concentrated layers occur at altitudes where atmospheric conditions are stable [22], where wind speeds and temperature are maximized, or at the highest altitude at which aerial temperatures allow for sustained flight [64].

Insect flight behaviour also includes offsetting drift. Migrant hoverflies and groups of Lepidoptera showed greater offsets in headings to compensate for wind-induced lateral drift [49,63]. Thus, migrants could correct for the difference between preferred and displacement direction, even though this lowered overall flight speeds [60,61]. Migratory flight behaviour in small insects such as aphids (aerial plankton) has remained less studied as they are difficult to extract from small-scale radars, and weather radars cannot separate individuals nor their behaviour. However, recent work with radar has shown that migrations of small insects like aphids are often more ‘active’ than previously thought: they decide when to actively enter the air or land and also exert some control over altitude, e.g. by producing lift in updrafts and ceasing to produce lift in downdrafts to remain neutrally buoyant [26,28].

Long-term monitoring of species EBVs traits provides an opportunity to understand how climate warming, changes in precipitation patterns, and shifting atmospheric conditions affect movement behaviour. While climate warming has advanced insect emergence phenology generally, consequences for insect migration phenology are as yet undescribed, and mismatches between shifts in the atmospheric 'engine’ of long-distance insect movement and the cues for migration may result in complex global change responses. A thorough understanding of insect flight behaviour and decision-making can also inform insect forecasting efforts, allowing for spatially explicit real-time predictions of insect movement. Integrating observations of insect position in the atmosphere with atmospheric dispersion models such as HySPLIT or NAME has been demonstrated to fit movement trajectories [65] and holds promise for pest monitoring systems.

### (c) Essential biodiversity variables class ‘community composition’

Researching community composition and species interactions is a challenging application of radar technology, as species identification is limited. Radars recognize diverse aerial targets and can differentiate well between biological and other targets, and usually also between birds and insects. However, the differentiation within groups of birds and insects depends upon additional morphological parameters and flight characteristics. However, even relatively coarse taxonomic resolution of species or size groups can provide information on the composition of aerial communities and their interactions. Currently, small-scale radars have greater use for this EBV class than weather radars because small-scale radars report on individual targets, resulting in greater taxonomic resolution. However, recent developments demonstrate the potential for taxonomic discrimination and species composition EBVs using weather radar as well as small-scale radar.

Small-scale radars use combinations of morphological features and wing beat frequencies to distinguish species or species groups. As many species overlap in their morphological parameters [66], additional reference information such as life history or aerial sampling data can improve taxonomic resolution. For example, in eastern Australia, high abundances of nocturnally migrating Australian plague locusts (*Chortoicetes terminifera*) are sufficiently common that size, shape and wingbeat frequency have been quantified and their signature can be recognized in entomological radar output [67,68].

Individual mass is the most important single feature differentiating insect radar signals [69], which has been used for echo selection [63] and to infer species identity in combination with aerial trapping [70]. Parameters related to body shape have been used to select echoes from morphologically distinct taxa such as ladybird beetles and hoverflies, particularly in systems where the composition of aerial taxa has been well-described. For example, observations of two hoverfly species were successfully extracted from a dataset of day-flying aerial migrants above the UK based on body mass and two principal ventral radar cross sections [51,61].

Machine learning approaches to differentiate insects based on a limited set of morphological parameters are rapidly improving and, e.g. allowed identifying 23 Lepidoptera and Odonata species in an aerial community in East China with a probability greater than 0.5 [69]. The importance of various characteristics in differentiating taxa depends on type and range of taxa being classified. In the above study, size was the most important parameter to differentiate between moth species, while wingbeat frequency could differentiate between orders with near complete certainty.

While identifying individual taxa is sometimes possible, radar-derived morphological parameters more often identify a target class with similar size, morphology and wingbeat frequency. For example, 6–10 distinct target classes were identified in an eight-month study in eastern Australia using small-scale radar [68]. Similarly, in a 14-year study of migrants in eastern Australia, clustering resulted in six target classes, with distinct seasonal patterns and interannual variation. While the classes were coarse (e.g. locusts, medium-sized moths), they form ecologically meaningful groupings and thus, could be used to derive ecological links between the groups, their similarities and differences in response to environmental parameters [66].

Although the use of radar to evaluate species interactions is overall still uncommon, some studies combined radar observations with other sampling to examine predator–prey interactions. For example, small-scale radar observations of two invasive ladybird species combined with suction trap data of their primary prey showed a temporal association between ladybird emigration flights and aphid abundance [71]. High-altitude flights in ladybirds increased when aphid abundances were low, suggesting that prey availability also drives long-distance flight behaviour. A unique example of species interactions on radar is that of insect-free patches, where a scan otherwise filled with nocturnal insects showed ‘gaps’, i.e. brief localized absence of insects, indicating evasive flights in response to ultrasonic signals from their bat predators [22].

Recent advances have been made using (dual-pol) weather radar to distinguish between biological scatterers. Gauthreaux & Diehl [72] showed how six types of biological scatterers (trans-gulf migrating birds, purple martins (*Progne subis*), waterfowl, free-tailed bats (*Tadarida brasiliensis*), broad-front movements of insects, and aquatic insect emergences (mayflies (Ephemeridae) and midges (Chironomidae)) vary from one another in their backscatter signals in dual-pol radar. Similarly, unsupervised classification algorithms based on dual-pol products and insects' use of airspace clustered insect observations in four distinct target classes. A comparison with ground sampling confirmed that the identified target classes were ecologically meaningful; i.e. the number of target classes observed on radar correlated well with macro-moth diversity on the ground [73]. While the taxonomic resolution is still coarse, differentiating broad groups of aerial taxa may also be useful in itself, e.g. for interactions between insects and insectivores [73].

Questions in the EBV class ‘species composition and interactions' have not received the attention in radar entomology they may deserve. Ongoing monitoring of this EBV class can provide insight into long-term changes in species composition in response to habitat loss, urbanization, nitrification or other global change drivers. For example, many insect communities are currently undergoing shifts in composition towards more generalist and pest species [5,74]. Quantifying such shifts in aerial taxa is possible through long-term radar monitoring efforts and improved target classification algorithms, providing insight in potential biodiversity loss.

### (d) Essential biodiversity variables class ‘ecosystem function’

Aerial insect movements and migrations provide a multitude of ecosystem functions by transporting genetic material, energy, biomass and nutrients across landscapes, and by being consumers or prey of other organisms [3]. Estimates of high-altitude insect flight indicate that, e.g. 3200 tons of insect biomass migrate annually above the southern UK [50]. A single emergence event of mayflies can transport 3078 tons of biomass from aquatic systems to the air, subsequently redistributing these nutrients and biomass across terrestrial and aquatic habitats [37], and in the Australian alps, migratory bogong moths (*Agrotis infusa*) annually deposit 7.2 metric tons of nitrogen (N; [75]). Besides transportation of biomass and nutrients, these movements impact human and natural systems through species interactions and ecosystem functioning.

Migratory insects transport pollen grains hundreds of kilometres during migration, facilitating gene flow and connecting otherwise isolated populations [51,76]. They function as prey resource in many systems; for example, the seasonal influx of bogong moths is prey for a range of species including aerial insectivores, ground dwelling mammals, and aquatic predators such as trout [75]. Similarly, Brazilian free-tailed bats (*T. brasiliensis*) adjust their foraging behaviour to feast on migratory moths such as fall armyworm (*Spodoptera frugiperda*), corn earworm *(Helicoverpa zea)* and cabbage looper (*Trichoplusia ni)* [77,78]. Migratory hoverfly species in the UK are important pollinators and also consume substantial quantities of other insects [51].

Studies that quantified insect movements also calculated their nutrient redistribution based on typical mass and nutrient composition of species/groups: the massive hoverfly migrations with a biomass of 30–80 tons of aerial biomass were estimated to result in a long-range transport of 1000– 2500 kg of N, 100–250 kg of phosphorus (P) and 50–150 GJ of energy, with southern Britain being a net exporter to continental Europe [51]. For all insect taxa measured in small-scale radar, an estimated 3200 tons of biomass moving annually over the southern UK involves 100 000 kg of N and 10 000 kg of P and 5.78 × 10^12^ Joules of energy [50]. Although southward and northward movements cancelled each other out over the 10-year study period, within specific years the net flux can vary in either direction, introducing temporal (within-year) fluctuations in local nutrient levels.

These insect ecosystem functions also constitute services and disservices that affect human agriculture, economy and health—the most relevant of which are probably crop consumption, crop pest consumption [51], and vectoring the agents of crop, livestock, wildlife and human diseases [79,80]. A prime example are locusts that are infamous worldwide for their capacity to devastate massive amounts of crop [81]. Consequently, attempts to automatically monitor mass immigrations, mass movements and outbreaks have long also been made with radars [82]. Although no fully automated monitoring and warning system for the immigration of pest insects is operational to date, various steps forward have been made: Australian plague locusts (*C. terminifera*) have been monitored locally with a network of insect monitoring radars [67] and more regionally with weather radar [83]; desert locusts (e.g. *Schistocerca gregaria* and other spp*.*) outbreaks have been detected by weather radars in India [82,84] and Argentina [85]. A pilot study tested the feasibility of a warning system for the immigration into Finland of important agricultural pests and vectors of crop diseases—bird-cherry aphids (*Rhopalosiphum padi*) and diamond-back moths (*Leptinotarsa decemlineata*)—using a combination of (polarimetric) weather radar, traps and dispersion modelling and numerical weather forecasts [45]. A similar study constructed emigration trajectories and identified source areas for known past infestations of noctuid cotton pests, demonstrating the potential to produce pest forecasts with high spatial and temporal precision for a crop advisory system [65].

Migrant insects also vector plant pathogens that can result in severe agricultural damages, e.g. barley yellow dwarf virus that is carried by bird-cherry aphids. Radars are particularly effective tools to study such pathogen transport because many vectors are small insects whose flight is predominantly wind-powered, and dependent on atmospheric conditions at flight altitude. Small-scale radars have been combined with aerial sampling to identify conditions for migratory flight and tested for the presence of rice ragged stunt virus in migrating planthoppers (*Nilaparvata lugens* [86]).

Another disservice by insect migrants is the vectoring of zoonotic parasites and pathogens. Vector-borne diseases are globally on the rise and constitute a disproportionate share among emerging infectious diseases [87]. Among vector-borne diseases, malaria is probably the most widely known and, despite decades of effort, still a massive health problem. The mosquitoes that transmit malaria (*Anopheles* spp*.*) also make sustained migratory flights over hundreds of kms in excessive abundances [88,89] over which they could transport parasites—yet, their movements have so far not been investigated with radars.

Given these economical and public health applications, (radar-based) automated monitoring and warning systems could assist in setting up timely and targeted mitigation measures: farmers and agricultural managers could confine pesticide use to areas and times when it would meaningfully reduce crop damage but avoid it for other times and areas, thereby greatly reducing the harmful effects of pesticides to other biodiversity and costs. Similarly, public health organizations could issue warnings locally or regionally and suggest the use of protective measures once major influxes of vectoring insects are expected. Radars can be an important element of such monitoring and warning systems but would best be complemented by trapping or other sorts of ‘ground-truthing’ and numeric modelling.

## 4. Synthesis—radars as biodiversity monitoring system for insects

Radar-based approaches are increasingly considered for monitoring aerial insect biodiversity and EBVs. The greatest advantages and unique selling points of radars are that they run autonomously, over medium to large spatial and temporal scales and are a non-invasive, non-destructive observation method, providing information on all aerial organisms [90]. Furthermore, the radar infrastructure is—at least for weather radars—already existing, and using their data for applications beyond meteorology would provide added societal value [91]. Their technology makes it inherently hard to identify species—a feature that has long precluded radars from the traditional biodiversity monitoring toolbox. However, ecological research increasingly depends on using disparate data sources (with unique strengths in spatial extent, taxonomic resolution and biases), providing an opportunity to integrate radar-based approaches with other monitoring approaches [90]. Radars can deliver unique information on insect EBVs that complements other approaches, and their use is expanding with promising current developments—from developments in radar technology that measure more features of aerial objects, machine learning and neural network algorithms that use these novel features to improve taxonomic classification, harmonization of data across radar systems and integration with other data sources, to efforts in providing data infrastructure for improved availability and access.

### (a) Radar hardware developments and classification algorithms

While small-scale radars keep on being developed further [92,93], developments in ‘frequency-modulated continuous wave' radars hold much promise for general insect biodiversity and biomass monitoring as they increase capabilities of taxonomic differentiation and extend the measurement range to much closer to the ground.

Weather radar infrastructure is undergoing key improvements with the deployment of dual-pol radars. Although dual-pol weather radars are not yet the standard setting worldwide, several countries have upgraded their entire weather radar fleet recently, e.g. the USA in 2013, Argentina in 2019, or are in the process of doing so, e.g. Australia, India, Europe. Dual-pol data products improve estimates of shape, size and variety of animals aloft, and have been a key innovation for ecological applications [34], particularly by improving target differentiation [72,73]. Other rapidly moving fields such as machine learning or convolutional neural networks will probably expand and improve the (automated) identification of insect and other aerial organisms from radar signals [94,95].

### (b) Combining data

The potential of radars for biodiversity monitoring can be enhanced by integrating data from different sources; both data from multiple radar systems and with data from other (monitoring) sources. The radar types introduced here are largely complementary in spatial and temporal coverage and taxonomic information and combining their data would yield more comprehensive and quantitatively different insights, e.g. small-scale radar can supplement the limited coverage of weather radar at low altitude or can provide data validation [31]. The strengths of small-scale radar—precise measurements of insects in a difficult to access habitat—can be supplemented with ground sampling of small insects not captured on radar to provide insight into insect movement patterns [50]. Similarly, the strengths of weather surveillance radar—existing networks with large spatial coverage—can be supplemented with historical data to provide insight into long-term variability in insect population dynamics [37]. Such combinations of radar data with data from other sources has already been successful in ornithological applications, e.g. weather radar observations of bird migration combined with acoustic monitoring and citizen science data [96,97]. Similar approaches in entomology could include citizen science sources, e.g. iNaturalist, computerized visual counts, acoustics, or molecular data.

### (c) Data infrastructure, availability and access

**Figure 3.**
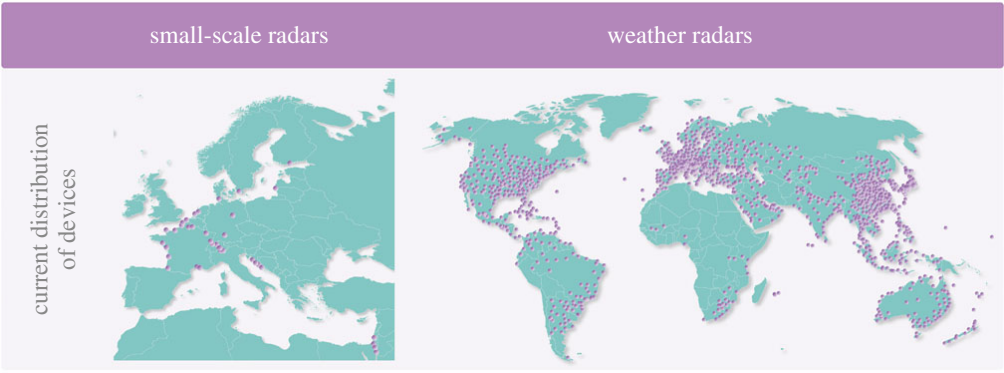
Distribution of small-scale and weather radars. Small-scale radars—here specifically BirdScan radars—(left panel) are mostly distributed in Europe (one device in the USA); weather radars (right panel) are widely distributed all over the world, although there are also larger gaps in regions without any.

Small-scale radars have mostly been used for dedicated biological/ecological purposes such as fundamental or applied research in local or regional settings. Most devices are run in Europe (figure 3), where an informal network of 17 small-scale radars has been set up recently [59] and only a handful more devices exist elsewhere in the world. Yet, as some models of small-scale radars have been developed into commercial off-the-shelf products, networks of small-scale radars could be set up nationally and internationally as standardized aerial biodiversity monitoring systems. Establishing such networks would require funding for the devices and the corresponding data infrastructure, which could (partly) be funded from offering services to various stakeholders, e.g. from wind energy, aviation safety or agriculture. Weather radars already exist in many places on the globe (figure 3) and are often organized in continental networks. However, whether their data are available for stakeholders other than meteorologists depends on national and international data sharing agreements and regulations. In many countries, radar data are not publicly available, and if available, the data are frequently stored in derivative format with a primary (and often: sole) focus on meteorological purposes, i.e. with biological ‘noise’ removed. However, progress is being made in providing access to data. In the USA, for instance, NEXRAD-data are publicly available through Amazon Web Services (AWS) and thus, accessible to anyone. AWS provides access to original resolution minimally processed radar scans (level II), stores a historical archive from 1991 to the present, and adds new scans within minutes of production, providing continuously updated near real-time radar data for other end-users such as the aeroecology community. The open access nature of NEXRAD radar data has catalysed non-meteorological radar-based research, fostering the development of R and Python packages to access the data and use it for biological studies [41,98].

In a similar effort in Europe, the European Network for the Radar Surveillance of Animal Migrations (www.enram.eu) has made a data-sharing agreement with OPERA that allows the scientific use of data. Also, the Australian Bureau of Meteorology has removed barriers to use of weather radar data by making the complete Australian operational radar archive (2000-current) available for research purposes [99,100]. Despite these efforts, data are still unavailable from many regions with weather radar coverage, but improved accessibility could potentially provide a wealth of biodiversity data for typically not-well monitored areas [101]. As a step towards using this potential of weather radars, the World Meteorological Organisation has recognized value of weather radar data beyond meteorology and recently adjusted their data policies (https://public.wmo.int/en).

Thus, radar-based approaches can be an important asset in the standardized biodiversity monitoring of the airspace—an essential habitat for a large proportion of the global biodiversity [102], which is poorly monitored and largely absent from legislation and policy despite its role being no less critical for biodiversity and ecosystem functioning than that of other habitats [12,13,102,103]. Radar data can provide abundances and biomass of aerial biodiversity [50], indicate the health of their populations, the intensity of interactions with resident communities, the magnitude of ecosystem functions provided [51] and, if changes over time and space are measured, even rough measures for demographic rates [104]. The greatest challenges for establishing weather- and small-scale radars as standardized monitoring system of the airspace are the currently scattered distribution of devices and data, the diverse data formats, software tools and lack of harmonization of radar—and, more importantly, their biodiversity products. The financial resources required to overcome these hurdles are, however, relatively low (compared to, e.g. meteorological monitoring by weather radars) and with the steps above, we have outlined the road map towards achieving this goal.

## Data Availability

This article has no additional data.

## References

[1] Satterfield DA, Sillett TS, Chapman JW, Altizer S, Marra PP. 2020 Seasonal insect migrations: massive, influential, and overlooked. Front. Ecol. Environ. **18**, 335-344. (10.1002/fee.2217)

[2] Chapman JW, Reynolds DR, Wilson K. 2015 Long-range seasonal migration in insects: mechanisms, evolutionary drivers and ecological consequences. Ecol. Lett. **18**, 287-302. (10.1111/ele.12407) 25611117

[3] Bauer S, Hoye BJ. 2014 Migratory animals couple biodiversity and ecosystem functioning worldwide. Science **344**, 1242552. (10.1126/science.1242552) 24700862

[4] Seibold S et al. 2019 Arthropod decline in grasslands and forests is associated with landscape-level drivers. Nature **574**, 671-674. (10.1038/s41586-019-1684-3) 31666721

[5] van Klink R, Bowler DE, Gongalsky KB, Swengel AB, Gentile A, Chase JM. 2020 Meta-analysis reveals declines in terrestrial but increases in freshwater insect abundances. Science **368**, 417-420. (10.1126/SCIENCE.AAX9931/SUPPL_FILE/AAX9931-VANKLINK-SM.PDF) 32327596

[6] Wagner DL. 2020 Insect declines in the anthropocene. Annu. Rev. Entomol. **65**, 457-480. (10.1146/annurev-ento-011019-025151) 31610138

[7] Kissling WD et al. 2018 Building essential biodiversity variables (EBVs) of species distribution and abundance at a global scale. Biol. Rev. **93**, 600-625. (10.1111/BRV.12359) 28766908

[8] Lumbierres M, Kissling WD. 2023 Important first steps towards designing the freshwater, marine and terrestrial essential biodiversity variable (EBV) workflows for the European biodiversity observation network. Res. Ideas Outcomes **9** e109120. (10.3897/RIO.9.E109120)

[9] Moersberger H et al. 2022 Europa biodiversity observation network: user and policy needs assessment. ARPHA Prepr. **3**, e84517. (10.3897/ARPHAPREPRINTS.E84517)

[10] Pereira HM et al. 2013 Essential biodiversity variables. Science **339**, 277-278. (10.1126/science.1229931) 23329036

[11] Bridge ES et al. 2011 Technology on the move: recent and forthcoming innovations for tracking migratory birds. Bioscience **61**, 689-698. (10.1525/bio.2011.61.9.7)

[12] Davy CM, Ford AT, Fraser KC. 2017 Aeroconservation for the fragmented skies. Conserv. Lett. **10**, 773-780. (10.1111/conl.12347)

[13] Chilson PB, Stepanian PM, Kelly JF. 2017 Radar aeroecology. In Aeroecology (eds PB Chilson, WF Frick, JF Kelly, F Liechti), pp. 277-309. Cham, Switzerland: Springer International Publishing. (10.1007/978-3-319-68576-2_12)

[14] Chilson PB, Bridge E, Frick WF, Chapman JW, Kelly JF. 2012 Radar aeroecology: exploring the movements of aerial fauna through radio-wave remote sensing. Biol. Lett. **8**, 698-701. (10.1098/rsbl.2012.0384) 22628093 PMC3440989

[15] Kelly JF, Shipley JR, Chilson PB, Howard K. W, Frick WF, Kunz TH. 2012 Quantifying animal phenology in the aerosphere at a continental scale using NEXRAD weather radars. Ecosphere **3**, art16. (10.1890/ES11-00257.1)

[16] Shamoun-Baranes J et al. 2014 Continental-scale radar monitoring of the aerial movements of animals. Mov. Ecol. **2**, 9. (10.1186/2051-3933-2-9)

[17] Chapman JW, Reynolds DR, Smith AD. 2003 Vertical-looking radar: a new tool for monitoring high- altitude insect migration. Bioscience **53**, 503. (10.1641/0006-3568(2003)053[0503:VRANTF]2.0.CO;2)

[18] Huuskonen A, Saltikoff E, Holleman I. 2014 The operational weather radar network in Europe. Bull. Am. Meteorol. Soc. **95**, 897-907.

[19] Bauer S et al. 2017 From agricultural benefits to aviation safety: realizing the potential of continent-wide radar networks. Bioscience **67**, 912-918. (10.1093/biosci/bix074) 29599538 PMC5862237

[20] Shamoun-Baranes J et al. 2021 Weather radars' role in biodiversity monitoring. Science **372**, 248 LP. (10.1126/science.abi4680) 33859027

[21] Long T, Hu C, Wang R, Zhang T, Kong S, Li W, Cai J, Tian W, Zeng T. 2020 Entomological radar overview: system and signal processing. IEEE Aerosp. Electron. Syst. Mag. **35**, 20-32. (10.1109/MAES.2019.2955575)

[22] Drake VA, Reynolds DR. 2012 Radar entomology: observing insect flight and migration. UK: CABI.

[23] Drake VA, Farrow RA. 1983 The nocturnal migration of the Australian plague locust, *Chortoicetes terminifera* (Walker) (Orthoptera: Acrididae): quantitative radar observations of a series of northward flights. Bull. Entomol. Res. **73**, 567-585. (10.1017/S0007485300009172)

[24] Noskov A, Achilles S, Bendix J. 2021 Presence and biomass information extraction from highly uncertain data of an experimental low-range insect radar setup. Diversity **13**,452. (10.3390/D13090452)

[25] Wainwright CE, Volponi SN, Stepanian PM, Reynolds DR, Richter DH. 2022 Using cloud radar to investigate the effect of rainfall on migratory insect flight. Methods Ecol. Evol. **14**, 655-668. (10.1111/2041-210X.14023)

[26] Wainwright CE, Stepanian PM, Reynolds DR, Reynolds AM. 2017 The movement of small insects in the convective boundary layer: linking patterns to processes. Sci. Rep. **7**, 1-8. (10.1038/s41598-017-04503-0) 28710446 PMC5511248

[27] Weisshaupt N, Hervo M, Haest B. 2023 Comparison of bird migration in a radar wind profiler and a dedicated bird radar. Remote Sens. Ecol. Conserv. **9**, 820-828. (10.1002/rse2.350

[28] Geerts B, Miao Q. 2005 Airborne radar observations of the flight behavior of small insects in the atmospheric convective boundary layer. Environ. Entomol. **34**, 361-377. (10.1603/0046-225X-34.2.361)

[29] Shi X, Schmid B, Tschanz P, Segelbacher G, Liechti F. 2021 Seasonal trends in movement patterns of birds and insects aloft simultaneously recorded by radar. Remote Sens. **13**, 1839. (10.3390/rs13091839)

[30] Drake VA, Chapman JW, Lim KS, Reynolds DR, Riley JR, Smith AD. 2017 Ventral-aspect radar cross sections and polarization patterns of insects at X band and their relation to size and form. Int. J. Remote Sens. **38**, 5022– 5044. (10.1080/01431161.2017.1320453)

[31] Nilsson C et al. 2018 Field validation of radar systems for monitoring bird migration. J. Appl. Ecol. **55**, 2552-2564. (10.1111/1365-2664.13174)

[32] Haest B et al. 2021 BirdScan community reference dataset. Zenodo. (10.5281/zenodo.5734961)

[33] Haest B, Hertner F, Schmid B, Preatoni D, De Groeve J, Liechti F. 2023 birdscanR: migration traffic rate calculation package for Birdscan MR1 radars. Zenodo. (10.5281/ZENODO.7665531)

[34] Stepanian PM, Horton KG, Melnikov VM, Zrnić DS, Gauthreaux SA. 2016 Dual-polarization radar products for biological applications. Ecosphere **7**, e01539. (10.1002/ecs2.1539)

[35] Nussbaumer R, Schmid B, Bauer S, Liechti F. 2021 A Gaussian mixture model to separate birds and insects in single-polarization weather radar data. Remote Sens. **13**, 1989. (10.3390/rs13101989)

[36] Westbrook JK, Eyster RS, Wolf WW. 2014 WSR-88D Doppler radar detection of corn earworm moth migration. Int. J. Biometeorol. **58**, 931-940. (10.1007/s00484-013-0676-5) 23748420

[37] Stepanian PM, Entrekin SA, Wainwright CE, Mirkovic D, Tank JL, Kelly JF. 2020 Declines in an abundant aquatic insect, the burrowing mayfly, across major North American waterways. Proc. Natl Acad. Sci. USA **117**, 2987-2992. (10.1073/pnas.1913598117) 31964842 PMC7022163

[38] Tielens EK, Cimprich PM, Clark BA, DiPilla AM, Kelly JF, Mirkovic D, Strand AI, Zhai M, Stepanian PM. 2021 Nocturnal city lighting elicits a macroscale response from an insect outbreak population. Biol. Lett. **17**, 20200808. (10.1098/rsbl.2020.0808) 33784873 PMC8086988

[39] Boulanger Y, Fabry F, Kilambi A, Pureswaran DS, Sturtevant BR, Saint-Amant R. 2017 The use of weather surveillance radar and high-resolution three dimensional weather data to monitor a spruce budworm mass exodus flight. Agric. For. Meteorol*.* **234–235**, 127-135. (10.1016/J.AGRFORMET.2016.12.018)

[40] Dokter AM, Liechti F, Stark H, Delobbe L, Tabary P, Holleman I. 2011 Bird migration flight altitudes studied by a network of operational weather radars. J. R. Soc. Interface **8**, 30-43. (10.1098/rsif.2010.0116) 20519212 PMC3024816

[41] Dokter AM et al*.* 2019 bioRad: biological analysis and visualization of weather radar data. Ecography (Cop.) **42**, 852-860. (10.1111/ECOG.04028)

[42] Gasteren HV, Holleman I, Bouten W, Loon EV, Shamoun-Baranes J. 2008 Extracting bird migration information from C-band Doppler weather radars. Ibis (Lond. 1859) **150**, 674-686. (10.1111/j.1474-919X.2008.00832.x)

[43] Kilambi A, Fabry F, Meunier V. 2018 A simple and effective method for separating meteorological from nonmeteorological targets using dual-polarization data. J. Atmos. Ocean. Technol. **35**, 1415-1424. (10.1175/JTECH-D-17-0175.1)

[44] Mirkovic D, Stepanian PM, Kelly JF, Chilson PB. 2016 Electromagnetic model reliably predicts radar scattering characteristics of airborne organisms. Sci. Rep. **6**, 35637. (10.1038/srep35637) 27762292 PMC5071894

[45] Leskinen M, Markkula I, Koistinen J, Pylkkö P, Ooperi S, Siljamo P, Ojanen H, Raiskio S, Tiilikkala K. 2011 Pest insect immigration warning by an atmospheric dispersion model, weather radars and traps. J. Appl. Entomol. **135**, 55-67. (10.1111/j.1439-0418.2009.01480.x)

[46] Dingle H. 2014 Migration: the biology of life on the move. Oxford, UK: Oxford University Press.

[47] Russell RW, May ML, Soltesz KL, Fitzpatrick JW. 1998 Massive swarm migrations of dragonflies (Odonata) in eastern North America. Am. Midl. Nat. **140**, 325-342. (10.1674/0003-0031(1998)140[0325:MSMODO]2.0.CO;2)

[48] Hawkes WLS et al. 2022 Huge spring migrations of insects from the Middle East to Europe: quantifying the migratory assemblage and ecosystem services. Ecography (Cop.) **2022**, e06288. (10.1111/ecog.06288)

[49] Chapman JW, Reynolds DR, Mouritsen H, Hill JK, Riley JR, Sivell D, Smith AD, Woiwod IP. 2008 Wind selection and drift compensation optimize migratory pathways in a high-flying moth. Curr. Biol. **18**, 514-518. (10.1016/j.cub.2008.02.080) 18394893

[50] Hu G, Lim KS, Horvitz N, Clark SJ, Reynolds DR, Sapir N, Chapman JW. 2016 Mass seasonal bioflows of high- flying insect migrants. Science **354**, 1584-1587. (10.1126/science.aah4379) 28008067

[51] Wotton KR, Gao B, Menz MHM, Morris RKA, Ball SG, Lim KS, Reynolds DR, Hu G, Chapman JW. 2019 Mass seasonal migrations of hoverflies provide extensive pollination and crop protection services. Curr. Biol. **29**, 2167-2173.e5. (10.1016/j.cub.2019.05.036) 31204159

[52] Chapman JW, Bell JR, Burgin LE, Reynolds DR, Pettersson LB, Hill JK, Bonsall MB, Thomas JA. 2012 Seasonal migration to high latitudes results in major reproductive benefits in an insect. Proc. Natl Acad. Sci. USA **109**, 14 924-14 929. (10.1073/pnas.1207255109) PMC3443120 22927392

[53] Stefanescu C *et* al. 2013 Multi-generational long-distance migration of insects: studying the painted lady butterfly in the Western Palaearctic. Ecography (Cop.) **36**, 474-486. (10.1111/j.1600-0587.2012.07738.x)

[54] Westbrook JK, Wolf WW, Allen S, Ward JD. 1998 NEXRAD Doppler weather radar network: potential for areawide surveillance of pest insect migrations. Proc. Beltwide Cott. Conf. **2**, 1304-1310.

[55] Tielens EK, Kelly J. 2024 Temperature, not net primary productivity, drives continental scale variation in insect flight activity. Phil. Trans. R. Soc. B **379**, 20230114. (10.1098/rstb.2023.0114) 38705173 PMC11070256

[56] Macgregor CJ, Williams JH, Bell JR, Thomas CD. 2019 Moth biomass has fluctuated over 50 years in Britain but lacks a clear trend. Nat. Ecol. Evol. **3**, 1645-1649. (10.1038/s41559-019-1028-6) 33990768

[57] Brower LP, Taylor OR, Williams EH, Slayback DA, Zubieta RR, Ramírez MI. 2012 Decline of monarch butterflies overwintering in Mexico: is the migratory phenomenon at risk? Insect Conserv. Divers. **5**, 95-100. (10.1111/j.1752-4598.2011.00142.x)

[58] Wood CR, Reynolds DR, Wells PM, Barlow JF, Woiwod IP, Chapman JW. 2009 Flight periodicity and the vertical distribution of high-altitude moth migration over southern Britain. Bull. Entomol. Res. **99**, 525-535. (10.1017/S0007485308006548) 19224662

[59] Haest B et al*.* 2024 Continental-scale patterns in diel flight timing of high-altitude migratory insects. Phil. Trans. R. Soc. B **379**, 20230116. (10.1098/rstb.2023.0116) 38705191 PMC11070267

[60] Chapman JW, Nilsson C, Lim KS, Bäckman J, Reynolds DR, Alerstam T. 2016 Adaptive strategies in nocturnally migrating insects and songbirds: contrasting responses to wind. J. Anim. Ecol. **85**, 115-124. (10.1111/1365-2656.12420) 26147535

[61] Gao B, Wotton KR, Hawkes WLS, Menz MHM, Reynolds DR, Zhai BP, Hu G, Chapman JW. 2020 Adaptive strategies of high-flying migratory hoverflies in response to wind currents. Proc. R. Soc. B **287**, 20200406. (10.1098/RSPB.2020.0406) PMC7341907 32486972

[62] Drake VA, Helm KF, Readshaw JL, Reid DG. 1981 Insect migration across Bass Strait during spring: a radar study. Bull. Entomol. Res. **71**, 449-466. (10.1017/S0007485300008476)

[63] Chapman JW, Nesbit RL, Burgin LE, Reynolds DR, Smith AD, Middleton DR, Hill JK. 2010 Flight orientation behaviors promote optimal migration trajectories in high-flying insects. Science **327**, 682-685. (10.1126/science.1182990) 20133570

[64] Wood CR, Chapman JW, Reynolds DR, Barlow JF, Smith AD, Woiwod IP. 2006 The influence of the atmospheric boundary layer on nocturnal layers of noctuids and other moths migrating over southern Britain. Int. J. Biometeorol. **50**, 193-204. (10.1007/S00484-005-0014-7/FIGURES/11) 16432728

[65] Westbrook JK, Eyster RS. 2017 Doppler weather radar detects emigratory flights of noctuids during a major pest outbreak. Remote Sens. Appl. Soc. Environ. **8**, 64-70. (10.1016/J.RSASE.2017.07.009)

[66] Hao Z, Drake VA, Taylor JR, Warrant E. 2020 Insect target classes discerned from entomological radar data. Remote Sens. **12**, 673. (10.3390/RS12040673)

[67] Drake VA, Wang H. 2013 Recognition and characterization of migratory movements of Australian plague locusts, *Chortoicetes terminifera*, with an insect monitoring radar. J. Appl. Remote Sens. **7**, 075095. (10.1117/1.JRS.7.075095)

[68] Drake VA. 2016 Distinguishing target classes in observations from vertically pointing entomological radars. Int. J. Remote Sens. **37**, 3811-3835. (10.1080/01431161.2016.1204028)

[69] Hu C, Kong S, Wang R, Zhang F. 2019 Radar measurements of morphological parameters and species identification analysis of migratory insects. Remote Sens. **11**, 1977. (10.3390/RS11171977)

[70] Chapman JW, Reynolds DR, Brooks SJ, Smith AD, Woiwood IP. 2006 Seasonal variation in the migration strategies of the green lacewing *Chrysoperla carnea* species complex. Ecol. Entomol. **31**, 378-388. (10.1111/j.1365-2311.2006.00797.x)

[71] Jeffries DL, Chapman J, Roy HE, Humphries S, Harrington R, Brown PMJ, Handley LJL. 2013 Characteristics and drivers of high-altitude ladybird flight: insights from vertical-looking entomological radar. PLoS ONE **8**, e82278. (10.1371/JOURNAL.PONE.0082278) 24367512 PMC3867359

[72] Gauthreaux S, Diehl R. 2020 Discrimination of biological scatterers in polarimetric weather radar data: opportunities and challenges. Remote Sens. **12**, 545. (10.3390/RS12030545)

[73] Lukach M et al. 2022 The development of an unsupervised hierarchical clustering analysis of dual-polarization weather surveillance radar observations to assess nocturnal insect abundance and diversity. Remote Sens. Ecol. Conserv. **8**, 698-716. (10.1002/RSE2.270) 36588588 PMC9790603

[74] Gossner MM, Menzel F, Simons NK. 2023 Less overall, but more of the same: drivers of insect population trends lead to community homogenization. Biol. Lett. **19**, 20230007. (10.1098/RSBL.2023.0007) 36987614 PMC10050920

[75] Green K. 2011 The transport of nutrients and energy into the Australian Snowy Mountains by migrating bogong moths *Agrotis infusa*. Austral. Ecol. **36**, 25-34. (10.1111/j.1442-9993.2010.02109.x)

[76] Coates JM, Keaney B, Scheele BC, Cunningham SA. 2023 Endangered bogong moths (*Agrotis infusa*) forage from local flowers after annual mass migration to alpine sites. Glob. Ecol. Conserv. **44**, e02482. (10.1016/J.GECCO.2023.E02482)

[77] Krauel JJ, Westbrook JK, Mccracken GF. 2015 Weather-driven dynamics in a dual-migrant system: moths and bats. J. Anim. Ecol. **84**, 604-614. (10.1111/1365-2656.12327) 25492132

[78] Krauel JJ, Brown VA, Westbrook JK, McCracken GF. 2018 Predator–prey interaction reveals local effects of high-altitude insect migration. Oecologia **186**, 49-58. (10.1007/s00442-017-3995-0) 29101468

[79] Reynolds DR, Chapman JW, Harrington R. 2006 The migration of insect vectors of plant and animal viruses. In Advances in virus research (ed. JM Thresh), pp. 453-517. New York, NY: Academic Press. 10.1016/S0065-3527(06)67012-7 17027687

[80] Reynolds DR, Chapman JW, Drake VA. 2017 Riders on the wind: the aeroecology of insect migrants. In Aeroecology (eds PB Chilson, WF Frick, JF Kelly, F Liechti), pp. 145-177. Cham, Switzerland: Springer International Publishing.

[81] Cressman, K.; Stefanski R. 2016 Weather and desert locusts. Geneva, Switzerland: World Meteorological Organization and Food and Agriculture Organization of the United Nations.

[82] Anjita NA, Indu J. 2023 Leveraging weather radars for desert locust monitoring. Remote Sens. Appl. Soc. Environ. **31**, 100983. (10.1016/J.RSASE.2023.100983)

[83] Rennie SJ. 2014 Common orientation and layering of migrating insects in southeastern Australia observed with a Doppler weather radar. Meteorol. Appl. **21**, 218-229. (10.1002/met.1378)

[84] Amarjyothi K, Kumar DP, Saikrishnan KC. 2022 Identification and tracking of locust swarms by Indian Doppler weather radar. IEEE Geosci. Remote Sens. Lett. **19**, 1-4. (10.1109/LGRS.2021.3086587)

[85] Poffo DA, Beccacece HM, Caranti GM, Comes RA, Drewniak ME, Martina A, Zapata AI, Rodriguez A, Saffe JN. 2018 Migration monitoring of *Ascia monuste* (Lepidoptera) and *Schistocerca cancellata* (Orthoptera) in Argentina using RMA1 weather radar. ISPRS J. Photogramm. Remote Sens. **145**, 340-348. (10.1016/J.ISPRSJPRS.2018.05.011)

[86] Riley JR, Smith AD, Reynolds DR. 2003 The feasibility of using vertical-looking radar to monitor the migration of brown planthopper and other insect pests of rice in China. Insect Sci. **10**, 1-19. (10.1111/j.1744-7917.2003.tb00359.x)

[87] Swei A, Couper LI, Coffey LL, Kapan D, Bennett S. 2020 Patterns, drivers, and challenges of vector-borne disease Emergence. Vector-Borne Zoonotic Dis. **20**, 159-170. (10.1089/vbz.2018.2432) 31800374 PMC7640753

[88] Dao A et al. 2014 Signatures of aestivation and migration in Sahelian malaria mosquito populations. Nature **516**, 387-390. (10.1038/nature13987) 25470038 PMC4306333

[89] Florio J et al. 2020 Diversity, dynamics, direction, and magnitude of high-altitude migrating insects in the Sahel. Sci. Rep. **10**, 1-14. (10.1038/s41598-020-77196-7) 33239619 PMC7688652

[90] van Klink R, Sheard JK, Høye TT, Roslin T, Do Nascimento LA, Bauer S. 2024 Towards a toolkit for global insect biodiversity monitoring. Phil. Trans. R. Soc. B **379**, 20230101. (10.1098/rstb.2023.0101) 38705179 PMC11070268

[91] Shamoun-Baranes J et al. 2022 Meteorological data policies needed to support biodiversity monitoring with weather radar. Bull. Am. Meteorol. Soc. **103**, E1234-E1242. (10.1175/BAMS-D-21-0196.1)

[92] Wang R, Zhang T, Cui K, Yu T, Jiang Q, Zhang R, Li J, Hu C. 2022 High-resolution and low blind range waveform for migratory insects’ taking-off and landing behavior observation. Remote Sens. **14**, 3034. (10.3390/RS14133034)

[93] Williams CR, Johnson KL, Giangrande SE, Hardin JC, Öktem R, Romps DM. 2021 Identifying insects, clouds, and precipitation using vertically pointing polarimetric radar Doppler velocity spectra. Atmos. Meas. Tech. **14**, 4425-4444. (10.5194/AMT-14-4425-2021)

[94] Chilson C, Avery K, McGovern A, Bridge E, Sheldon D, Kelly J. 2019 Automated detection of bird roosts using NEXRAD radar data and convolutional neural networks. Remote Sens. Ecol. Conserv. **5**, 20-32. (10.1002/RSE2.92)

[95] Belotti MCTD et al*.* 2023 Long-term analysis of persistence and size of swallow and martin roosts in the US Great Lakes. Remote Sens. Ecol. Conserv. **9**, 469-482. (10.1002/RSE2.323)

[96] Van Doren BM, Lostanlen V, Cramer A, Salamon J, Dokter A, Kelling S, Bello JP, Farnsworth A. 2022 Automated acoustic monitoring captures timing and intensity of bird migration. J. Appl. Ecol. **60**, 433-444. (10.1111/1365-2664.14342)

[97] Rosenberg K V. et al*.* 2019 Decline of the North American avifauna. Science **366**, 120-124. (10.1126/SCIENCE.AAW1313) 31604313

[98] Helmus JJ, Collis SM. 2016 The Python ARM radar Toolkit (Py-ART), a library for working with weather radar data in the Python programming language. J. Open Res. Softw. **4**, 25. (10.5334/JORS.119)

[99] Rogers RM, Buler JJ, Wainwright CE, Campbell HA. 2020 Opportunities and challenges in using weather radar for detecting and monitoring flying animals in the Southern Hemisphere. Austral. Ecol. **45**, 127-136. (10.1111/AEC.12823)

[100] Soderholm J, Protat A, Jakob C. 2019 Australian operational weather radar level 1 dataset. Electron. Dataset. (10.25914/508X-9A12)

[101] Shi X, Hu C, Soderholm J, Chapman J, Mao H, Cui K, Ma Z, Wu D, Fuller RA. 2023 Prospects for monitoring bird migration along the East Asian-Australasian Flyway using weather radar. Remote Sens. Ecol. Conserv. **9**, 169-181. (10.1002/rse2.307)

[102] Lambertucci SA, Shepard ELC, Wilson RP. 2015 Human-wildlife conflicts in a crowded airspace. Science **348**, 502-504. (10.1126/science.aaa6743) 25931541

[103] Diehl RH. 2013 The airspace is habitat. Trends Ecol. Evol. **28**, 377-379. (10.1016/j.tree.2013.02.015) 23506968

[104] Dokter AM, Farnsworth A, Fink D, Ruiz-Gutierrez V, Hochachka WM, La Sorte FA, Robinson OJ, Rosenberg K V., Kelling S. 2018 Seasonal abundance and survival of North America's migratory avifauna determined by weather radar. Nat. Ecol. Evol. **2**, 1603-1609. (10.1038/s41559-018-0666-4) 30224817

